# The association of serum total bile acid with new-onset hypertension during pregnancy

**DOI:** 10.1186/s12884-022-05211-y

**Published:** 2022-11-26

**Authors:** Weinan Deng, Lizi Zhang, Qiting Du, Yulian Li, Jingsi Chen, Lili Du, Dunjin Chen

**Affiliations:** 1 Department of Obstetrics and Gynecology, Guangdong Provincial Key Laboratory of Major Obstetric Diseases, Guangdong Engineering and Technology Research Center of Maternal-Fetal Medicine, Guangdong-Hong Kong-Macao Greater Bay Area Higher Education Joint Laboratory of Maternal-Fetal Medicine, The Third Affiliated Hospital of Guangzhou Medical University, Guangzhou, PR China; 2 The Medical Center for Critical Pregnant Women in Guangzhou, The Third Affiliated Hospital of Guangzhou Medical University, 63 Duobao Road, Liwan District, Guangzhou, 510150 China

**Keywords:** New-onset hypertension, Preeclampsia, Total bile acid, Risk factors, Intrauterine growth retardation, Low birth weight

## Abstract

**Background:**

There has been considerable interest in the interrelationship between the liver and hypertension. The relationship between serum total bile acid (TBA) and hypertension has been reported. Moreover, intrahepatic cholestasis of pregnancy was correlated to gestation hypertension. However, the association between maternal serum TBA level in the normal range and new-onset hypertension disorders during pregnancy remains unclear. The present study aimed to evaluate the relationship between maternal serum TBA level in the normal range and the risk, disease severity and adverse pregnancy outcomes of new-onset hypertension during pregnancy.

**Method:**

Using the electronic medical records on all pregnant women from the Department of Obstetrics and Gynecology, Third Affiliated Hospital of Guangzhou Medical University, between 2014 and 2020, we conducted a retrospective cohort study of 2581 singleton pregnant women with maternal serum TBA levels in the normal range. Patients were grouped into the non-hypertension during pregnancy (1071), gestational hypertension (480) and preeclampsia (1030) groups.

**Result:**

We found that maternal serum TBA levels were significantly higher in the preeclampsia and gestational hypertension groups than in the non-hypertension group (*p* < 0.01). Multiple logistic regression analysis showed that TBA level was independently and significantly associated with preeclampsia and gestational hypertension (odds ratio: 1.37, 95% confidence interval [CI]: 1.27–1.48, *p* = 0.001, odds ratio: 1.34, 95% confidence interval [CI]: 1.24–1.46, *p* = 0.005, respectively). Moreover, elevated TBA level was positively associated with the risk of severe PE and negatively with mild PE (*p* < 0.01). In addition, maternal serum TBA levels were negatively related to birth weight (*p* < 0.001).

**Conclusions:**

These results suggest that maternal serum TBA in the normal range also might be a valuable biomarker for disease severity in preeclampsia and gestational hypertension. Additionally, our results also indicate associations of serum total bile acid levels in the normal range with an increased risk of fetal growth restriction and low birth weight among offspring. These results suggest that TBA could serve as a prognostic biomarker for new-onset hypertension during pregnancy.

## Background

Hypertensive disorders of pregnancy (HDP) are common gestational complications that cause 10–15% of maternal deaths every year worldwide [1]. HDP includes chronic hypertension, preeclampsia (PE) with chronic hypertension and new-onset hypertension, including gestational hypertension (GH) or PE [2]. Among these, PE is one of the most important components and is defined by systolic blood pressure (SBP) over 140 mmHg and/or diastolic blood pressure (DBP) over 90 mmHg combined with urine protein over 0.3 g/24 h [3]. PE has a worldwide incidence of approximately 3–5% [4]. HDP threatens maternal and fetal health and increases the risk of long-term disease. Many studies report that the risk of cardiovascular diseases among HDP patients is 2 times higher than that among normal pregnant women, and the prevalence of chronic hypertension is 1.5 times higher among HDP patients [5]. Children born to mothers with PE have an even higher incidence of long-term cardiovascular diseases [6].

Recently, there has been more interest in the interrelationship between liver dysfunction and hypertension. The liver, the center of metabolism, has complicated functions, including synthesis and secretion of proteins, biotransformation of biomolecules, carbohydrate metabolism, lipid metabolism and bile acid metabolism [7]. Numerous enzymes are synthesized in the liver, and many of these enzymes have been reported to be markers for evaluating liver health. Moreover, some of them, such as aspartate aminotransferase (AST), alanine aminotransferase (ALT), and γ-glutamyltransferase (GTT), have been reported to be positively associated with hypertension [8] and could assist in the diagnosis of hypertension.

The synthesis and secretion of bile acid (BA) is one of the most important functions of the liver. BA has been found to be a part of the digestive system over the last few decades and is important for chyle digestion and absorption of lipid-soluble vitamins [9, 10]. However, increasing evidence shows that BA can be a signaling molecule that participates in the occurrence of many diseases, including hypertension [11, 12]. The incidence of PE among patients with intrahepatic cholestasis of pregnancy (ICP) is approximately 8–25%, which is much higher than that in normal pregnancy (1.8–4.4%) [13–15], suggesting that BA might be associated with PE. Among all PE patients, approximately 10–20% have hemolysis, elevated liver enzymes and low platelet (HELLP) syndrome [16]. A previous case report of four women diagnosed with ICP and severe HELLP syndrome suggested that BA might be associated with both the occurrence and development of PE [17]. However, the relationship between maternal serum TBA and HDP remains unclear, especially with total bile acid (TBA) levels in the normal range. The primary purpose of this study was to demonstrate the association of maternal serum TBA in the normal range with new-onset hypertension and pregnancy outcomes.

## Method

### Data collection

**Fig. 1:**
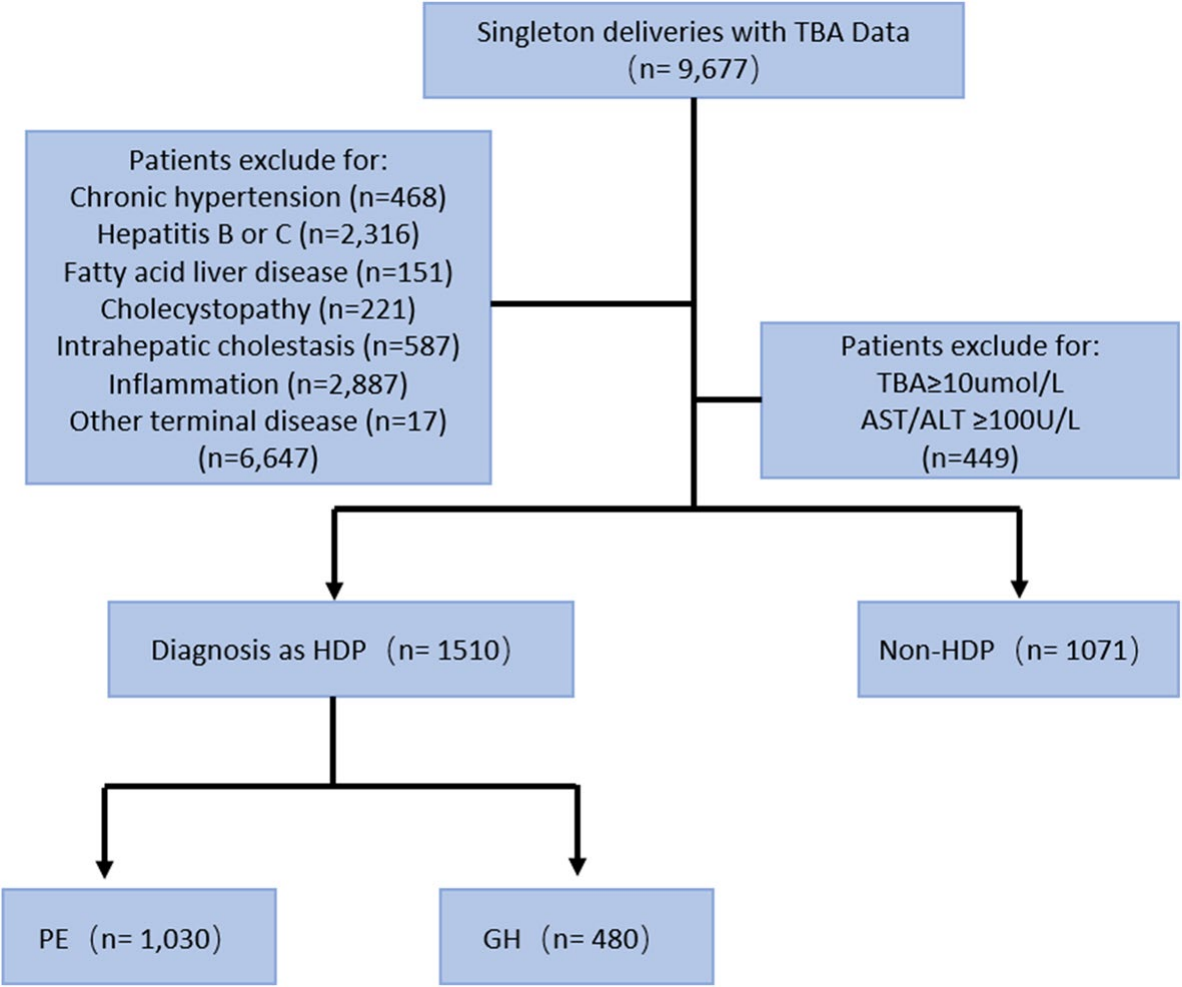
Study population flowchart

As shown in Fig. 1, we collected data from women who underwent routine gestational care from 2014 to 2020 at the Third Affiliated Hospital of Guangzhou Medical University. There were 9,677 singleton deliveries with TBA tests, and patients with chronic hypertension, hepatitis B or C, cholecystopathy, inflammation, a diagnosis of ICP, serum TBA levels ≥ 10 µmol/L, other terminal diseases such as phase iv cancer, end-stage renal disease, and AST or ALT levels over 100 U/L were excluded. A total of 1071 women were included in the non-HDP group, and 1510 women were included in the HDP group and further divided into PE (*n* = 1030) and GH (*n* = 480) subgroups.

Maternal clinical parameters were collected from medical record, including maternal age, gestational age at delivery (GA), body mass index (BMI), systolic blood pressure (SBP), diastolic blood pressure (DBP), whether receive in-vitro fertilization (IVF), nulliparity, pregestational diabetes infant birth weight and body length. SBP and DBP data in HDP group were collected for the first time measured after diagnosis, and in the non-HDP group, SBP and DBP were collected for the first time measured after hospitalized. Maternal fasting blood samples were collected by first-time examination after hospitalization, after an overnight fast of at least 8 h at a mean gestational age of 20 to 34 weeks. The biomarkers included TBA, ALB, TC, TG, AST, ALT, T bilirubin, and 24-h proteinuria. TBA, TC, TG, AST, ALT were assessed by Roche cobas^@^8000 modular analyzer series (Mannheim, BW, Germany), and T bilirubin, ALB and 24-h proteinuria Roche cobas^@^ C501(Mannheim, BW, Germany).

The criteria suggested by the American College of Obstetricians and Gynecologists (ACOG) for the diagnosis of pregnancy-associated hypertension were adopted. PE was defined as systolic blood pressure ≥ 140 mmHg or diastolic blood pressure ≥ 90 mmHg over at least two intervals of 6 h after 20 weeks of gestation with previously normal blood pressure and new-onset proteinuria (> 0.3 g/24-h or at least > 1 + on protein dipstick when urine could not be collected for 24 h). And liver function impaired, thrombocytopenia, severe persistent right upper quadrant or epigastric pain and not accounted for by alternative diagnoses, renal insufficiency, pulmonary edema, new onset headache unresponsive to acetaminophen and visual disturbances should diagnosed as PE for women with gestational hypertension in the absence of proteinuria [3]. Severe PE was defined as systolic blood pressure ≥ 160 mmHg or diastolic blood pressure ≥ 110 mmHg and new-onset headache or Visual disturbances. Early-onset PE (EOPE) was defined as development PE before 34 weeks of gestation and late-onset PE (LOPE) was defined as development PE after 34 weeks of gestation [18].

The primary pregnancy outcomes in our study were low birth weight and fetal growth restriction (FGR). FGR was diagnosed following the guidelines for FGR [19], as indicated by ultrasound-estimated fetal weight or circumference of the abdomen smaller than 10% of the birth population. Low birth weight (LBW) was defined as birth weight less than 2500 g.

### Statistical analysis

Data were presented as the mean ± standard deviation or median (25–75 percentile). According to the normality of the variable distributions, measurement data were expressed as the means ± standard deviations, except for GA, SBP, DBP, TBA, AST, ALT, 24 h proteinuria and birth length. An independent-samples t-test was performed for age, BMI, ALB, TC, TG and birth weight. Count data were expressed as proportions (%), and the Mann–Whitney U test was performed for GA, SBP, DBP, TBA, AST, ALT, 24 h proteinuria and birth length. Spearman correlation analysis was used to evaluate the correlation between maternal serum TBA levels and birth weight and between AST/ALT and TBA levels. Multiple logistic regression analysis was performed to examine the influence of the following variables: maternal age, prepregnancy BMI, AST, ALT, T bilirubin, TC, TG, ALB, pregestational diabetes, nulliparity and IVF. The odds ratios with 95% CIs were calculated.

All statistical analyses were performed with IBM SPSS Statistics version 20 software (IBM Corp., Chicago, IL, USA) and GraphPad Prism 6 (GraphPad Software, Inc); *p* values < 0.05 were considered indicative of statistical significance.

## Result

After ineligible patients were excluded, 2581 pregnant women were included for subsequent analysis. 480 women were diagnosed with GH, 1030 were diagnosed with PE, and 1071 pregnant women were non-HDP. The characteristics of the pregnant women among these groups were shown in Table 1. Various clinical parameters were compared between non-HDP and HDP groups. HDP patients gave birth to newborns with significantly lower birth weight (*P* < 0.01) and an earlier gestational age at delivery than the non-HDP patients (interquartile range (IQR) of GA: 39 (38–40) vs. 38 (37–39), 37 (34–38), for non-HDP, GH and PE, respectively). Similar to other reports, HDP patients tended to have a higher BMI (*P* < 0.01), AST and ALT levels (*p* < 0.01). TC and TG levels were significantly higher in the PE group (*p* < 0.01) than in the non-HDP group but not in the GH group. Smoking and drinking were considered as risk factors for HDP [20]; however, possibly because of cultural reasons, in our study, only one patient smoked and one drank.

**Table 1:** Clinical and biochemical characteristics of the study patients

Parameter	**Non-HDP**	**GH**	*p value* ^a^	**PE**	*p value* ^b^
Patients (n)	1071	480		1030	
Age (years)	31.64 ± 5.15	32.9 ± 4.76	0.5	32.39 ± 4.89	0.514
GA (weeks)	39 (38–40)	38 (37–39)	< 0.001	37 (34–38)	< 0.01
Prepregnancy BMI (kg/m2)	25.01 ± 4.32	25.58 ± 4.08	0.004	24.92 ± 4.14	0.001
Birth weight (g)	3068.97 ± 666.34	2912.66 ± 747.11	< 0.001	2506.07 ± 933.74	< 0.001
Body length (cm)	49 (47–51)	49 (47–51)	< 0.001	47 (43–50)	< 0.001
Lifestyle					
Drinking (n,%)	1 (0.09)	0 (0)		0 (0)	
Smoking (n,%)	1 (0.09)	0 (0)		0 (0)	
Radiation exposure (n,%)	1 (0.09)	0 (0)		3 (0.29)	
Blood pressure					
SBP (mmHg)	109.98 ± 7.85	126.35 ± 24.42	< 0.001	141.10 ± 24.93	< 0.001
DBP (mmHg)	70.87 ± 6.5	83.84 ± 13.23	< 0.001	84.58 ± 16.96	< 0.001
Biochemical indicators					
TBA (µmol/L)	1.8 (1.18–2.7)	2.1 (1.5–3.2)	< 0.001	2.2 (1.5–3.4)	< 0.001
ALB (g/L)	36.19 ± 4.39	35.82 ± 4.38	< 0.001	34.33 ± 5.55	< 0.001
TC (µmol/L)	4.95 ± 1.77	5.1 ± 1.75	< 0.001	5.46 ± 1.63	0.002
TG (µmol/L)	2.54 ± 1.45	2.7 ± 1.39	0.802	3.05 ± 1.6	< 0.001
AST (U/L)	14.4 (12.0–17.9)	14.6 (12.5–19.9)	0.89	16 (12.4–20.1)	< 0.001
ALT (U/L)	8.05 (6.2–11.4)	8.95 (6.9–13.38)	< 0.001	9.8 (6.75–14.2)	< 0.001
T bilirubin (µmol/L)	4.2 (3.22–6.39)	4.15 (3.1–5.38)	0.258	3.8 (2.65–5.39)	< 0.001
24 h-proteinuria	0.32 (0.24–0.55)	0.27 (0.18–0.54)	0.766	0.55 (0.31–2.18)	< 0.001

Values are expressed as the mean ± standard deviation, number, or median (Q1–Q3); *GA* gestational age at delivery, *TBA* total bile acid, *ALB* albumin; *AST* aspartate aminotransferase, *ALT* alanine aminotransferase, *T bilirubin* total bilirubin, *TC* total cholesterol, *TG* triglyceride, *SBP* systolic blood pressure, *DBP* diastolic blood pressure

^a^*p*-value for GH compared with non-HDP

^b^*p*-value for PE compared with non-HDP

**Fig. 2:**
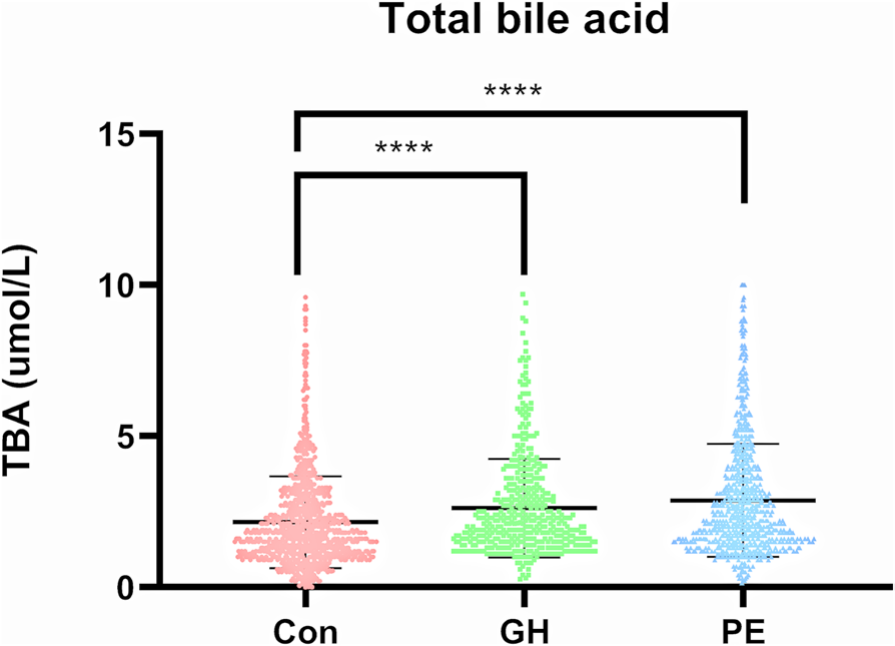
Maternal serum level at 20–34 weeks of pregnancy. **** *p* < 0.001

In our study, the mean serum level of TBA was 2.2 (1.5–3.4) µmol/L for the GH patients and 2.1 (1.5–3.2) µmol/L for the PE patients, which was approximately 1.3-fold higher than that in the non-HDP group (1.8 (1.18–2.7) µmol/L) (Fig. 2) and 1.5-fold higher than the previously reported value of 1.5 ± 1.1 µmol/L in normal pregnancy [21]. The serum TBA levels were significantly correlated with AST and ALT (*ρ* = 0.8, *p* < 0.01, *ρ* = 1.58, *p* < 0.01, respectively, data not shown) in pregnant women, which is consistent with a previous report [22].

**Fig. 3:**
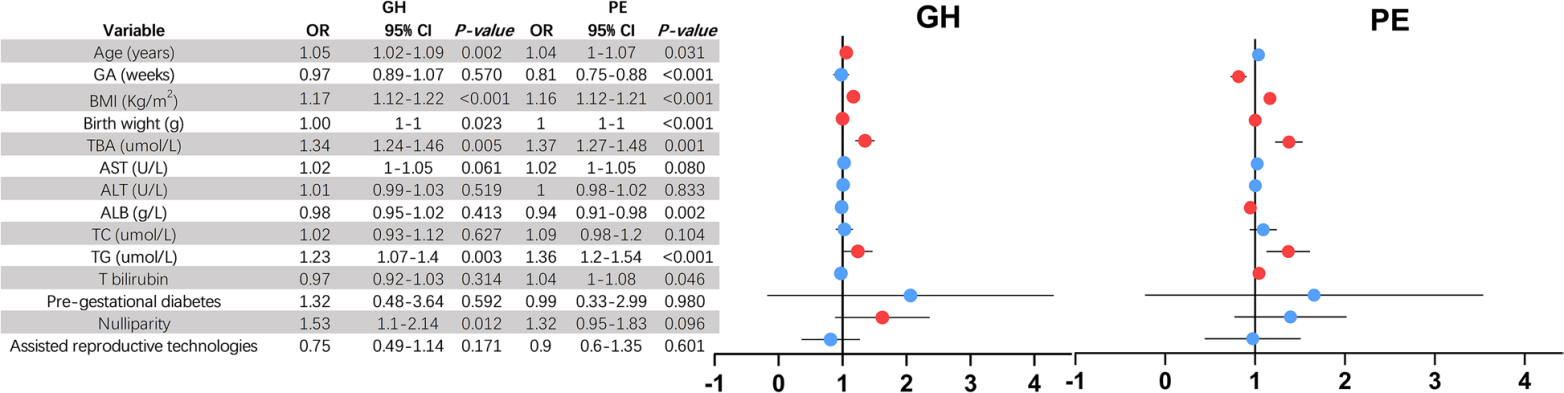
Multiple logistic regression analysis of factors for new-onset hypertension during pregnancy. The red dots represent *p* < 0.05. PE: preeclampsia, GH: gestational hypertension, OR: odds ratio, 95% CI: 95% confidence intervals, GA: gestational age at delivery, TBA: total bile acid, ALB: albumin, AST: aspartate aminotransferase, ALT: alanine aminotransferase, T bilirubin: total bilirubin, TC: total cholesterol, TG: triglyceride, SBP: systolic blood pressure, DBP: diastolic blood pressure

Multivariate logistic regression analysis was used to test whether serum TBA was significantly and independently correlated with PE and GH (Fig. 3). As in previous reports, age, BMI, pregestational diabetes, nulliparity and IVF were risk factors for PE [4], these factors were adjusted for Multivariate logistic regression analysis. After adjustment for high-risk factors, GA, BMI, AST, ALT, T bilirubin, TC, TG and ALB, the serum TBA was an important independent factor associated with PE and GH (odds ratio with PE: 1.37, 95% confidence interval [CI]: 1.27–1.48, *P* = 0.001, odds ratio with GH: 1.34, 95% confidence interval [CI]: 1.24–1.46, *P* = 0.005, respectively); beyond that, TG was also an independent factor associated with PE and GH (odds ratio: 1.36, 95% confidence interval [CI]: 1.2–1.54, *p* < 0.001, odds ratio: 1.23, 95% confidence interval [CI]: 1.07–1.4, *p* = 0.003, respectively).

**Fig. 4:**
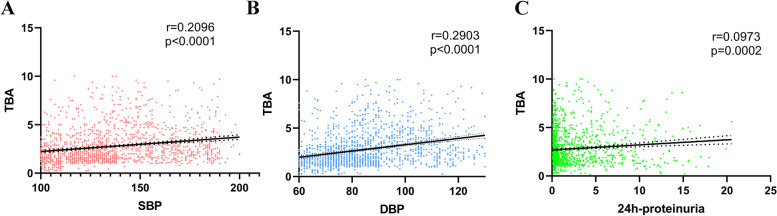
Correlation of maternal serum TBA with (**A**) SBP, (**B**) DBP and (**C**) 24 h-proteinuriaby using Spearman’s correlation

The diagnosis of PE was based on blood pressure and proteinuria. Thus, we asked if maternal serum TBA level was associated with systolic blood pressure, diastolic blood pressure and 24 h-proteinuria. We apply Spearman correlation analysis between serum TBA and SBP, DBP, 24 h-proteinuria in patients that diagnosed for HDP. As shown in Fig. 4, maternal serum TBA was positively associated with SBP (*r* = 0.2096, *p* < 0.0001), DBP (*r *= 0.2903, *p* < 0.0001) and 24 h-proteinuria (*r* = 0.0937, *p* = 0.0002).

Next, we tested the relationship between serum TBA and the severity of PE. Based on a previous report [23], we categorized TBA levels into 4 groups. As shown in Table 2, higher serum TBA levels were associated with increased severity of PE. Compared with total pregnant women, higher TBA levels showed more percentage of PE patients. Using a serum TBA level less than 1.6 µmol/L as the reference, patients with serum TBA levels between 1.6 to 4.1 µmol/L (OR: 1.82, CI: 1.47–2.26), higher than 4.1 µmol/L and less than 8.2 µmol/L (OR: 2.37; CI: 1.71–3.28) and between 8.2 µmol/L and 10 µmol/L (OR: 4.63; CI: 1.89–11.35) were at higher risk for PE. For early-onset PE (EOPE), TBA levels between 1.6 to 4.1 µmol/L (OR: 1.62, CI: 1.23–2.12), higher than 4.1 µmol/L and less than 8.2 µmol/L (OR: 1.93; CI: 1.33–2.81) and between 8.2 µmol/L and 10 µmol/L (OR: 3.46; CI: 1.32–9.04) had higher risks of EOPE. And for late-onset PE (LOPE), TBA levels between 1.6 to 4.1 µmol/L (OR: 1.45, CI: 1.15–1.82), higher than 4.1 µmol/L and less than 8.2 µmol/L (OR: 1.63; CI: 1.17–2.28) and between 8.2 µmol/L and 10 µmol/L (OR: 3.75; CI: 1.57–8.98) carry the highest risk for LOPE.

**Table 2:** Patients in different degree of TBA level

**TBA**	**No**	**PE**			**EOPE**			**LOPE**			**Mild PE**			**Severe PE**		
		**No. (%)**	***p value***^**a**^	**OR (95% CI)**	**No. (%)**	***p value***^**a**^	**OR (95% CI)**^**a**^	**No. (%)**	***p value***^**a**^	**OR (95% CI)**^**a**^	**No. (%)**	***p value***^**b**^	**OR (95% CI)**	**No. (%)**	***p value***^**b**^	**OR (95% CI)**
** < 1.6**	865	270 (31.25)		1(reference)	112(12.95)		1(reference)	158(18.27)		1(reference)	147 (54.44)		1(reference)	123 (45.56)		1(reference)
**1.6 to < 4.1**	1322	545 (41.26)	< 0.001	1.82(1.47–2.26)	241(18.23)	0.001	1.62(1.23–2.12)	304(23.0)	0.002	1.45(1.15–1.82)	237 (43.49)	0.002	0.61(0.45–0.83)	308 (56.51)	0.002	1.64(1.2–2.24)
**4.1 to < 8.2**	361	192 (53.33)	< 0.001	2.37(1.71–3.28)	93(25.76)	0.001	1.93(1.33–2.81)	99(27.42)	0.004	1.63(1.17–2.28)	74 (38.54)	0.005	0.56(0.38–0.84)	118 (61.46)	0.005	1.78(1.19–2.66)
**8.2 to < 10**	37	23 (63.89)	0.001	4.63(1.89–11.35)	10(27.03)	0.011	3.46(1.32–9.04)	13(35.14)	0.003	3.75(1.57–8.98)	5 (21.74)	0.007	0.23(0.08–0.67)	18 (78.26)	0.007	4.27(1.48–12.3)

Adjustment for AST, ALT, T bilirubin, TC, TG, ALB, pregestational diabetes, nulliparity and IVF

*PE* preeclampsia, *EOPE* early-onset PE, *LOPE* late-onset PE

^a^Compared PE patients with non-PE patients in total pregnant women

^b^Compared mild PE or severe PE with all PE patients

Preeclampsia is divided into mild preeclampsia and sever preeclampsia according to the severity. Among PE patients, increased serum TBA levels showed more percentage of severe PE. TBA levels between 1.6 to 4.1 µmol/L (OR: 1.64, CI: 1.2–2.24), higher than 4.1 µmol/L and less than 8.2 µmol/L (OR: 1.78; CI: 1.19–2.66) and between 8.2 µmol/L and 10 µmol/L (OR: 4.27; CI: 1.48–12.3) had higher risks of severe PE. And for mild PE, TBA levels between 1.6 to 4.1 µmol/L (OR: 0.61, CI: 0.45–0.83), higher than 4.1 µmol/L and less than 8.2 µmol/L (OR: 0.56; CI: 0.38–0.84) and between 8.2 µmol/L and 10 µmol/L (OR: 0.23; CI: 0.08–0.67) (Table 3).

**Table 3:** LBW and FGR patients in different degree of TBA level

**TBA**	**No**	**LBW**			**IUGR**		
		**No. (%)**	***p value***^**a**^	**OR (95% CI)**	**No. (%)**	***p value***^**b**^	**OR (95% CI)**
** < 1.6**	404	201 (49.75)		1(reference)	84 (20.79)		1(reference)
**1.6 to < 4.1**	817	428 (52.39)	0.15	1.199(0.937–1.535)	165 (20.20)	0.21	1.97(1.72–2.31)
**4.1 to < 8.2**	261	184 (70.50)	< 0.001	2.65(1.89–3.72)	75 (28.74)	0.01	2.44(1.99–3.07)
**8.2 to < 10**	28	20 (71.43)	0.01	3.11(1.32–7.33)	9 (32.14)	0.04	2.81(1.79–4.15)

Adjustment for maternal age, prepregnancy BMI, AST, ALT, T bilirubin, TC, TG, ALB, pregestational diabetes, nulliparity and IVF

^a^Compared between LBW patients and non-LBW patients in HDP patients

^b^Compared between FGR patients and non- FGR patients in HDP patients

**Fig. 5:**
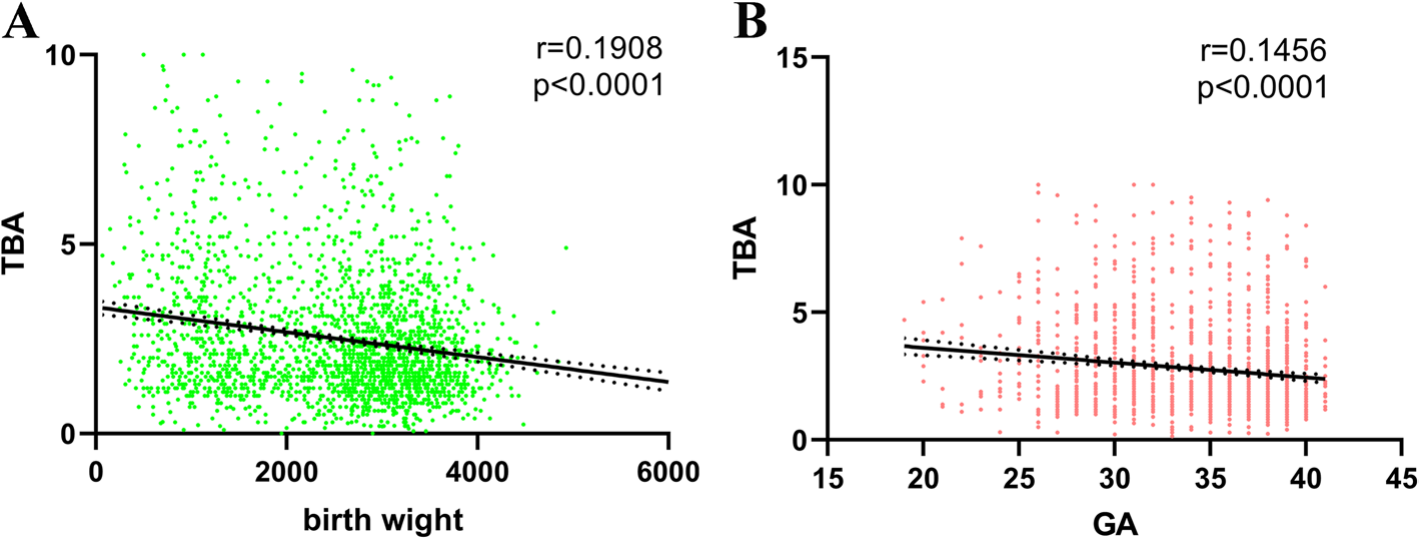
Correlation of the serum TBA level in HDP patients with (**A**) birth weight and (**B**) gestation weeks of delivery by using Spearman’s correlation

PE patients have a higher risk of low birth weight (LBW), fetal growth restriction (FGR), and earlier GA. Thus, we performed Spearman correlation to test the relationship of maternal serum TBA and GA as well as birth weight in HDP patients. The results showed that TBA was significantly correlated with birth weight (*r* = 0.1908, *p* < 0.0001) and GA (*r* = 0.1456, *p* < 0.0001) (Fig. 5).

Then, we tested the numbers of patients of LBW and FGR in different TBA degrees. In HDP patients, the TBA level was positively related with LBW and FGR. Using a serum TBA level less than 1.6 µmol/L as the reference, serum TBA levels higher than 4.1 µmol/L and less than 8.2 µmol/L (OR: 2.65; CI: 1.89–3.72) and between 8.2 µmol/L and 10 µmol/L (OR: 3.11; CI: 1.32–7.33) carry a higher risk for LBW. Pregnancies with serum TBA levels higher than 4.1 µmol/L and less than 8.2 µmol/L (OR: 2.44; CI: 1.99–3.07) and between 8.2 µmol/L and 10 µmol/L (OR: 2.81; CI: 1.79–4.15) had a higher risk for FGR (Table 3).

### Comment

This study had the following findings: 1) Maternal serum TBA was significantly and positively associated with HDP and severity of PE; 2) The TBA level was negatively related to birth weight and FGR. Taken together, these findings indicate that maternal serum TBA is a potential prognostic biomarker of PE.

Intrahepatic cholestasis of pregnancy (ICP) was determined by pruritus occurring during pregnancy and serum TBA above 10 µmol/L, and excluding for elevated serum aminotransferase and/or serum bile acids related to other causes of liver test abnormalities [24, 25]. ICP has been reported to be related to maternal and fetal diseases during pregnancy, such as PE, gestational diabetes mellitus (GDM) [26], placental abnormalities [27] and stillbirth [28]. However, the relationship between HDP and TBA levels in the normal range remains unclear.

In our study, we demonstrate that maternal serum TBA levels in the normal range were significantly higher in the HDP group than those in the non-HDP group and could be an independent risk factor for HDP. In addition, the level of serum TBA is significantly and positively associated with the severity of PE. Although, total bile acid was not significant change during pregnancy, especially second and third trimesters [29, 30]. High levels of bile acid have been reported to cause placental structural damage due to vasoconstriction of the chorionic plate veins [31] and affect the placental antioxidant system, which leads to oxidative stress and increases placental syncytial knots [32]. An increase in the number of placental syncytial knots was reported in PE [27]. Furthermore, although the pathophysiology of PE is not fully understood, increased maternal endoglin and soluble-fms-like tyrosine kinase-1 (s-Flt1) levels are known to be major contributors to PE. Oxidative stress was reported to increase the expression of s-Flt1 by trophoblast cells of the placenta [33]; thus, increased serum bile acid-induced oxidative stress might upregulate the expression of s-Flt1 and could therefore explain our observation. Moreover, we found maternal TBA level was positively related to SBP, DBP and proteinuria. There were studies that reported that bile acid had a vasodilation effect [34, 35]. These studies are more focused on the secondary bile acid, especially Ursodeoxycholic acid (UDCA) and lithocholic acid (LCA), which were predominant in liver cirrhosis. However, other bile acid derivates, such as cholic acid, have less effect on vasodilation, and marinobufagenin (MGB) elevated blood pressure by inhibiting vascular NA/K-ATPase [36, 37].

Fetal complications, especially LBW and FGR, have been reported in PE and ICP pregnancies. A recent study in China reported increased TBA levels with a decrease in birth weight [38]. Song and coworkers also found that serum TBA levels in all ranges were associated with the risk of FGR and that HDP had an additive effect on the association [24]. Consistent with these studies, our data show that the maternal TBA level was negatively associated with birth weight and positively associated with the risk of LBW and FGR. Besides, previous reports more focused on the relationship of LBW and FGR with all range TBA or even TBA levels in ICP patients [24, 38]. Based on our study, even TBA level in the normal range, the elevation is also positively related to the risk of LBW and FGR. Bile acid accumulation may affect placental vascular remodeling and thus cause insufficient placental perfusion, leading to fatal nutritional deficiency and FGR [39, 40]. Furthermore, toxic bile acid disrupts the expression of placental angiogenic and antiangiogenic factors, including s-Flt1 and endoglin, which have been reported to contribute to PE and FGR [41]. The oxidative stress caused by bile acid accumulation increases the expression of proinflammatory cytokines and chemokines, such as TNF-α, C-reactive protein and IL-8, in the placenta and maternal peripheral organs [42], which will activate the NF-κB pathway. Recent studies have revealed that placentas from FGR infants show strong Nf-κB p65 immunoreactivity [43], and animal experiments have also found maternal inflammation and oxidative stress in FGR [44]. Further studies are required to elucidate the pathological mechanism by which excessive bile acid causes FGR, especially in PE patients.

To our knowledge, this is the first study to investigate the relationship between HDP and maternal serum TBA levels in the normal range. In addition, we included hospitalized patients, which ensured the integrity of the information and allowed us to study the relationship between HDP and TBA. However, the present study has some limitations. First, this study is a single-center study. Second, because this study is a cross-sectional observational study, causality cannot be determined. Third, although blood sample were collected after overnight fasting, we do not measure hormone level, dietary habit and emotional status that are factors that can influence the TBA level. Last, bile acid has many secondary forms; in this study, we discuss only the total bile acid in serum.

## Conclusion

In conclusion, the present study demonstrates that maternal serum TBA can be an indicator of new-onset HDP, especially PE. Moreover, an increasing level of TBA can be a cost-effective predictor for PE, which is associated with both clinical severity and pregnancy outcomes. Therefore, maternal serum TBA has the potential to be a biomarker for PE and to facilitate disease management.

## Data Availability

The datasets generated and/or analysed during the current study are not publicly available due to the confidentiality of patient information but are available from the corresponding author on reasonable request.

## Abbreviations

TBA: Total bile acid
BA: Bile acid
HDP: Hypertensive disorders of pregnancy
PE: Preeclampsia
GH: Gestational hypertension
GA: Gestational age at delivery
BMI: Body mass index
IVF: In-vitro fertilization
ALB: Albumin
AST: Aspartate aminotransferase
ALT: Alanine aminotransferase
T bilirubin: Total bilirubin
TC: Total cholesterol
TG: Triglyceride
SBP: Systolic blood pressure
DBP: Diastolic blood pressure
HELLP: Hemolysis, elevated liver enzymes and low platelet
LBW: Low birth weight
FGR: Fetal growth restriction
EOPE: Early-onset PE
LOPE: Late-onset PE
ICP: Intrahepatic cholestasis of pregnancy
GDM: Gestational diabetes mellitus
IQR: Interquartile range
OR: Odds ratio
95% CI: 95% Confidence intervals
MGB: Marinobufagenin
UDCA: Ursodeoxycholic acid
LCA: Lithocholic acid
s-Flt1: Soluble-fms-like tyrosine kinase-1
